# Three New *Escherichia coli* Phages from the Human Gut Show Promising Potential for Phage Therapy

**DOI:** 10.1371/journal.pone.0156773

**Published:** 2016-06-09

**Authors:** Marion Dalmasso, Ronan Strain, Horst Neve, Charles M. A. P. Franz, Fabien J. Cousin, R. Paul Ross, Colin Hill

**Affiliations:** 1 School of Microbiology, University College Cork, Cork, Ireland; 2 APC Microbiome Institute, University College Cork, Cork, Ireland; 3 Department of Microbiology and Biotechnology, Max Rubner-Institut, Kiel, Germany; 4 Teagasc Biotechnology Centre, Moorepark Food Research Centre, Fermoy, Co. Cork, Ireland; ContraFect Corporation, UNITED STATES

## Abstract

With the emergence of multi-drug resistant bacteria the use of bacteriophages (phages) is gaining renewed interest as promising anti-microbial agents. The aim of this study was to isolate and characterize phages from human fecal samples. Three new coliphages, ɸAPCEc01, ɸAPCEc02 and ɸAPCEc03, were isolated. Their phenotypic and genomic characteristics, and lytic activity against biofilm, and in combination with ciprofloxacin, were investigated. All three phages reduced the growth of *E*. *coli* strain DPC6051 at multiplicity of infection (MOI) between 10^−3^ and 10^5^. A cocktail of all three phages completely inhibited the growth of *E*. *coli*. The phage cocktail also reduced biofilm formation and prevented the emergence of phage-resistant mutants which occurred with single phage. When combined with ciprofloxacin, phage alone or in cocktail inhibited the growth of *E*. *coli* and prevented the emergence of resistant mutants. These three new phages are promising biocontrol agents for *E*. *coli* infections.

## Introduction

Several members of the *Escherichia coli* species have emerged as important human pathogens. For example, *E*. *coli* O157:H7 can be the cause of acute diarrhea, hemorrhagic colitis and hemolytic uremic syndrome [1]. Shiga toxin-producing *E*. *coli* (STEC) and enteropathogenic *E*. *coli* (EPEC) are of particular concern, as they cause infections which can be fatal in sensitive populations [2, 3]. The emergence of multi-drug resistant bacterial strains is increasing [4] and alternative treatments to antibiotics are urgently required.

Bacteriophages (phages), viruses of bacteria, have been exploited for their potential as antibacterial agents since their discovery over 100 years ago [5, 6]. Some phage preparations, such as ListShield^™^ and LISTEX^™^ P100, are now approved for the control of pathogenic bacteria in food [7, 8]. The veterinary use of phage as feed additives to prevent *E*. *coli* infection in animals is currently being examined. For example, pigs infected with enterotoxigenic *E*. *coli* (ETEC) showed more resistance to diarrhea due to ETEC infection when treated with phages than non-phage treated animals [9]. While phages have been extensively used in some countries of Eastern Europe for clinical purposes, there is still no approved phage treatment in the Western world [8]. Recently it has been shown that phages efficiently reduced the number of enteroaggregative *E*. *coli* cells in the gut of mice [10]. Some studies have established the safety of T4 phage cocktails applied orally in humans, establishing that they do not cause adverse effects on host health and that they do not affect the commensal microbiota [11, 12]. By discovering new phages effective against pathogenic bacteria, it is possible to develop alternative treatment methods either for food safety, veterinary or clinical use.

The aim of this study was to isolate and characterize new bacteriophages from the human gut against *E*. *coli*, and to investigate their antibacterial properties alone and in cocktail.

## Material and Methods

### Strains, phage isolation from faecal samples, and host range determination

The strains used in this study have previously been characterized and are kept in the collections of Moorepark Food Research Centre (Ireland) and University College Cork (Ireland) (Table 1). Thirty frozen faecal samples, 20 from community patients and 10 from patients in long-term care facilities, from the ELDERMET project [13], were used to isolate phages. Ethical approval was provided by the Cork Clinical Research Ethics Committee. The samples used in this study have been described in a previous publication [14]. Briefly, 0.5 g of sample were suspended in 10 ml sterile saline magnesium buffer (100 mM NaCl, 8 mM MgSO_4_, 50 mM Tris [pH 7.5], and 0.002% [wt/vol] gelatin), mixed by vortexing for 10 min and centrifuged twice at 2500×g for 10 min at 4°C. The supernatant was then filtered sterile with 0.45 μm syringe filters. 100 μl of filtrate were mixed with 100 μl of an overnight culture of the target *E*. *coli* strain (DPC6009, DPC6050, DPC6051, DPC6054, DPC6055, E2348/68) (Table 1), before mixing with 3 ml of LB agar (0.5% agar, Oxoid, Basingstoke, United kingdom) containing 10 mM MgSO_4_, and poured on top of a LB agar plate. The plates were incubated at 37°C until formation of plaques. Phages ɸAPCEc01, ɸAPCEc02, and ɸAPCEc03 all got strain DPC6051 as primary host. Host range determination was performed using spot tests using a methodology previously described [15].

**Table 1:** Strains used in this study and host range of phages ɸAPCEc01, ɸAPCEc02 and ɸAPCEc03.

				Phage host range		
Strain	Strain designation	Description	Reference	ɸAPCEc01	ɸAPCEc02	ɸAPCEc03
*E*. *coli*	042	EAEC O44:H18, patient with diarrhea	[16]	-	-	-
*E*. *coli*	2348/69	EPEC O127:H6, outbreak of infantile diarrhea	[17]	-	-	-
*E*. *coli*	O157:H7 Sakai Δshx (RIMD 0509952)	EHEC O157:H7, Sakai outbreak	[18]	-	-	+
*E*. *coli*	H10407	ETEC O78:H11:K80, adult with cholera-like symptoms	[19]	-	-	+
*E*. *coli*	HM605	Colonic mucosal isolate from a Crohn’s disease patient	[20]	-	-	-
*E*. *coli*	MG1655			-	+	+
*E*. *coli*	A0 34/86	O83:K24:H31, commensal porcine isolate	[21]	-	-	-
*E*. *coli*	Nissle			-	-	+
*E*. *coli*	UTI89	Patient with cystitis	[22]	-	-	-
*E*. *coli*	CFT073	Pyelonephritis isolate	[23]	-	-	-
*E*. *coli*	DPC6009	NA		+	+	+
*E*. *coli*	DPC6050	NA		+	+	+
*E*. *coli*	DPC6051	NA		+	+	+
*E*. *coli* O157:H7	DPC6054	Non toxigenic		-	+	+
*E*. *coli* O157:H7	DPC6055	Non toxigenic		-	-	+
*E*. *coli* EPEC	E2348/68	NA		-	-	-
*Shigella sonnei*		Patient with shigellosis		+	-	-
*Salmonella* Typhimurium	DPC6046	Pig carcass swab	[24]	-	-	-
*Salmonella* Typhimurium	DPC6452	Faecal sample		-	-	-

^+^ Presence of a clear lytic zone

^-^ Absence of lytic zone

NA: Not available

As strain DPC6051 was the host used to isolate the three phages, it was used in all the assays presented in this study.

### One-step growth curve

One-step growth experiments were performed in triplicate to assess the burst size, and latency and rise phases of phages ɸAPCEc01, ɸAPCEc02, and ɸAPCEc03 using a method previously described [25] with the following modifications. Incubations were performed in LB broth supplemented with 10 mM MgSO_4_, at 37°C. A multiplicity of infection (MOI) of about 0.001 was used. The burst size was calculated by using the following formula, where “titer after burst” is the phage titer after the initial burst and “phage added” is the phage titer added before adsorption (7.8 × 10^4^ pfu/ml, 1.6 × 10^4^ pfu/ml and 4.2 × 10^4^ pfu/ml for phages ɸAPCEc01, ɸAPCEc02, and ɸAPCEc03, respectively): burst size = (titer after burst—titer at T0)/(phage added—titer at T0) [25].

### Bacterial challenge tests and phage activity against *E*. *coli* biofilms

Bacterial challenge tests and biofilm assays were performed in 96-well plates, in triplicate. For both tests, each condition was tested in 8 wells of the plate, containing 100 μl of diluted culture and 100 μl of diluted phage lysate. Phage lysate dilutions were performed in phage buffer (20 mM Tris-HCl [pH 7.2], 10 mM NaCl, 20 mM MgSO_4_). When using a combination of the three phages, each single phage was added in equal proportions to create the cocktail. The positive control wells contained only the bacterial culture and 100 μl of phage buffer.

For the bacterial challenge tests, the microplate was filled with an overnight culture of *E*. *coli* DPC6051 strain diluted in 2× LB broth containing 20 mM MgSO_4_ to obtain cell numbers of 10^4^ cfu/ml. This is of particular interest in the context of food decontamination where pathogenic bacteria are present at low concentrations. The tested conditions were phage MOI’s values ranging from 10^−3^ to 10^4^. The plate was incubated at 37°C for 24 h. The optical density (OD_600nm_) was measured in each well at the end of incubation.

For the biofilm assays, 20 ml of LB broth were inoculated with 200 μl of an overnight culture of strain DPC6051, and were used to fill the microplate, before being incubated at 37°C for 24 h to allow the bacterial cells to adhere to the wells. After removing the liquid culture without disturbing the cells attached to the wells as for the rest of the procedure, the wells were washed with PBS, and filled with 100 μl of 2× LB broth containing 20 mM MgSO_4_ and 100 μl of lysate dilutions. The plates were incubated at 37°C up to 48 h. The tested conditions were phage concentrations ranging from 10^2^ to 10^9^ phages per well. A colorimetric assay with XTT containing menadione was performed as previously described [26, 27], to assess the metabolic activity of the bacterial cells in the biofilm after phage treatment. Briefly, the biofilms were gently washed with phosphate buffered saline, then 100 μL of a solution containing 500 mg XTT/L (2,3-bis[2-methyloxy-4-nitro-5-sulfophenyl]-2H-tetrazolium-5-carboxanilide) (Sigma) and 50 μM menadione (Sigma) was added to each well. After a 2 h-incubation in the dark, the absorbance was measured at 492 nm using a microtiter plate reader (Molecular Devices Spectramax M3, Sunnyvale CA, USA). A Student’s t-test was performed to assess the significance of the phage action for both tests (p<0.05).

### Phage and antibiotic combination against *E*. *coli*

Combinations of phage (MOI of 1) and 4 μg/ml of ciprofloxacin HCl (MIC of 5 μg/ml) (Santa-Cruz Biotechnology, Dallas, USA) were tested in triplicate against a growing culture of strain DPC6051 (8.1 ± 0.04 log cfu/ml), in 5 ml LB supplemented with 10 mM MgSO_4_, and incubated at 37°C for 24 h. Ciprofloxacin is an antibiotic of the fluoroquinolone class, that inhibits bacterial replication by acting on DNA gyrases.

A miniaturized enumeration method previously described [28] was performed to evaluate the frequency of antibiotic-resistant mutants after phage-antibiotic treatment. The threshold of detection was 20 cfu/ml.

### Transmission electron microscopic (TEM) analysis

Phage lysates were purified on a cesium chloride gradient by ultracentrifugation [25], and 100 μl were dialyzed against phage buffer for 20 min on 0.025 μm VSWP membrane filters (Merck Millipore). Negative staining of phages and transmission electron microscopic analysis were performed as previously described [29].

### DNA extraction and genome sequencing

DNA was extracted from the cesium chloride purified fractions as previously described [25]. DNA samples were sent to GATC (Germany) for whole phage genome sequencing using an Illumina HiSeq 2500 sequencer with 2x100 bp read length. The reads generated by the Illumina instrument were assembled at GATC.

### *In silico* analysis of the genomes

Protein-encoding open reading frames (ORFs) were predicted using Glimmer [30]. Initial functional annotation of the ORFs and percentage amino acid identities were determined using BLASTP[31] and RAST server [32]. Transfer RNAs (tRNAs) were screened in the genomes using ARAGORN [33].

The BRIG software [34] was used for genome comparison of phage genomes which gave the best hit scores using BLASTP. Consequently, ɸAPCEc01 genome was compared to *E*. *coli* phages T4, vB_EcoM_JS09, RB27, vB_EcoM-VR7, Av-05, HX01, vB_EcoM_PhAPEC2, and RB69 (GenBank numbers NC_000866, NC_024124, NC_025448, NC_014792, KM190144, NC_018855, NC_024794, and NC_004928, respectively), and to *Shigella* phage Shf125875 (GenBank KM407600). ɸAPCEc02 genome was compared to *E*. *coli* phages rv5, 2 JES-2013, vB_EcoM_FFH2, and vB_EcoM-FV3 (GenBank numbers NC_011041, NC_022323, NC_024134, and NC_019517, respectively). ɸAPCEc03 genome was compared to *E*. *coli* phages T5, bV_EcoS_AKFV33, DT57C, DT571/2, and vB_EcoS_FFH1 (GenBank numbers NC_005859, NC_017969, KM979354, KM979355, and KJ190157, respectively), to *Salmonella* phages SPC35, Shivani, and Stitch (GenBank numbers HQ406778, KP143763, and KM236244, respectively), and to *Yersinia* phage phiR201 (GenBank HE956708).

### Accession numbers

The complete genome sequences of ɸAPCEc01, ɸAPCEc02 and ɸAPCEc03 have been deposited in GenBank under accession numbers KR422352, KR698074 and KR422353, respectively.

## Results and Discussion

### Phage morphology

The human gut is a natural reservoir of numerous phages with promising antibacterial activities [35]. Three new *E*. *coli* phages, ɸAPCEc01, ɸAPCEc02, and ɸAPCEc03, were isolated from three different human faecal samples of elderly patients, all in long-term care with no known health disorder [36]. The samples from which the phages were isolated did not display remarkable bacterial traits compared to the other samples. The percentages of *E*. *coli*/*Shigella* were 2.08%, 0.07% and 0.24% in the samples where ɸAPCEc01, ɸAPCEc02, and ɸAPCEc03 were isolated respectively [36].

**Fig 1:**
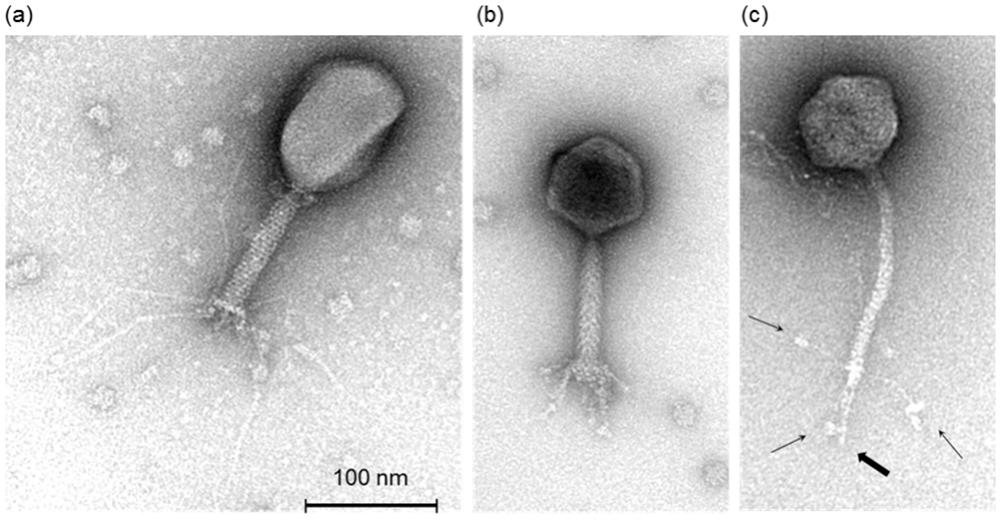
Transmission electron micrographs of *E*. *coli* phages ɸAPCEc01 (a), ɸAPCEc02 (b), and ɸAPCEc03 (c). The thin arrows in micrograph c indicate the 3 flexible fibres attached to the distal end of the phage tail. The terminal baseplate spike in c is illustrated by the thick arrow.

TEM analysis showed that phages ɸAPCEc01 and ɸAPCEc02 have contractile tails characteristic of the *Myoviridae* family (Fig 1a and 1b). Phage ɸAPCEc01 has a prolate head, and six thin bent tail fibres and short tail spikes attached below the baseplates (Fig 1a). The morphological characteristics and dimensions of phage ɸAPCEc01 (Table 2) are in accordance with T4 phage morphology [37]. Phage ɸAPCEc02 has an isodiametric head, and six short tail fibres attached to the baseplate (Fig 1b). Its morphological characteristics and dimensions (Table 2) are consistent with that of rV5-like phages [38, 39]. Phage ɸAPCEc03 has an isometric head, a flexible non-contractile tail, a characteristic distal tail spike, and three flexible bent fibres with distal globular structures (Fig 1c). Phage ɸAPCEc03 morphology and dimensions (Table 2) are characteristic of T5 phage from the *Siphoviridae* family [40].

**Table 2:** Dimensions of the three *E*. *coli* phages isolated in this study.

	ɸAPCEc01	ɸAPCEc02	ɸAPCEc03
Family	*Myoviridae*	*Myoviridae*	*Siphoviridae*
Head morphology	Prolate	Isometric	Isometric
Head dimensions^a^	118.5 ± 3.3 [n = 12]^b^ x 84.8 ± 5.1 [n = 13]	Ø 82.6 ± 4.6 [n = 8]	Ø 78.8 ± 3.7 [n = 11]
Tail length^a^	112.2 ± 1.8 [n = 13]	119.0 ± 3.7 [n = 7]	188.8 ± 5.5 [n = 10]
Tail width^a^	20.9 ± 1.2 [n = 13]	20.3 ± 1.3 [n = 7]	12.0 ± 1.5 [n = 9]
Tail fibre length^a^	152.0 ± 4.8 [n = 11]	46.0 ± 3.4 [n = 8]	53.0 ± 2.1 [n = 9]
Tail spike length^a^	15.5 ± 2.1 [n = 15]		43.6 ± 5.6 [n = 6]

^a^ Dimensions are expressed in nm

^b^[number of measurements]

### Host range

Phage ɸAPCEc03 had the broadest host range and targeted 9 out of the 16 *E*. *coli* strains tested, including an *E*. *coli* O157:H7 strain, an enterohaemorrhagic *E*. *coli* (EHEC) strain and an ETEC strain (Table 1). This broad host range makes phage ɸAPCEc03 an interesting candidate as a biocontrol agent, for example along the farm-to-fork chain, where *E*. *coli* is one of the most frequently encountered food pathogens [41], or for clinical purposes for treating *E*. *coli* infections [42, 43]. Phage ɸAPCEc02 targeted five *E*. *coli* strains, including an *E*. *coli* O157:H7 strain (Table 1). Interestingly, ɸAPCEc01 targeted three *E*. *coli* strains and a *Shigella sonnei* strain (Table 1). *S*. *sonnei* is responsible for diarrheal disease in industrialized countries and more recently in developing countries [44]. A siphophage infecting these two species has also been reported [45]. The fact the ɸAPCEc01 can target two bacterial genera is an asset of this phage for potential use as antimicrobial agent. In all cases, a higher number of appropriate strains should be tested in order to assess the full range of action of the three phages before considering their future application as antimicrobial agents. It is also necessary to use cocktails of multiple phages due to the great diversity of *E*. *coli* strains and thus to overcome the narrow host range of some phages. Besides, other methods for testing phage host range, like efficiency of plating on secondary hosts, could also be performed to avoid false positive in the case of lysis from without phenomenon [46, 47].

### Phage population dynamics

**Fig 2:**
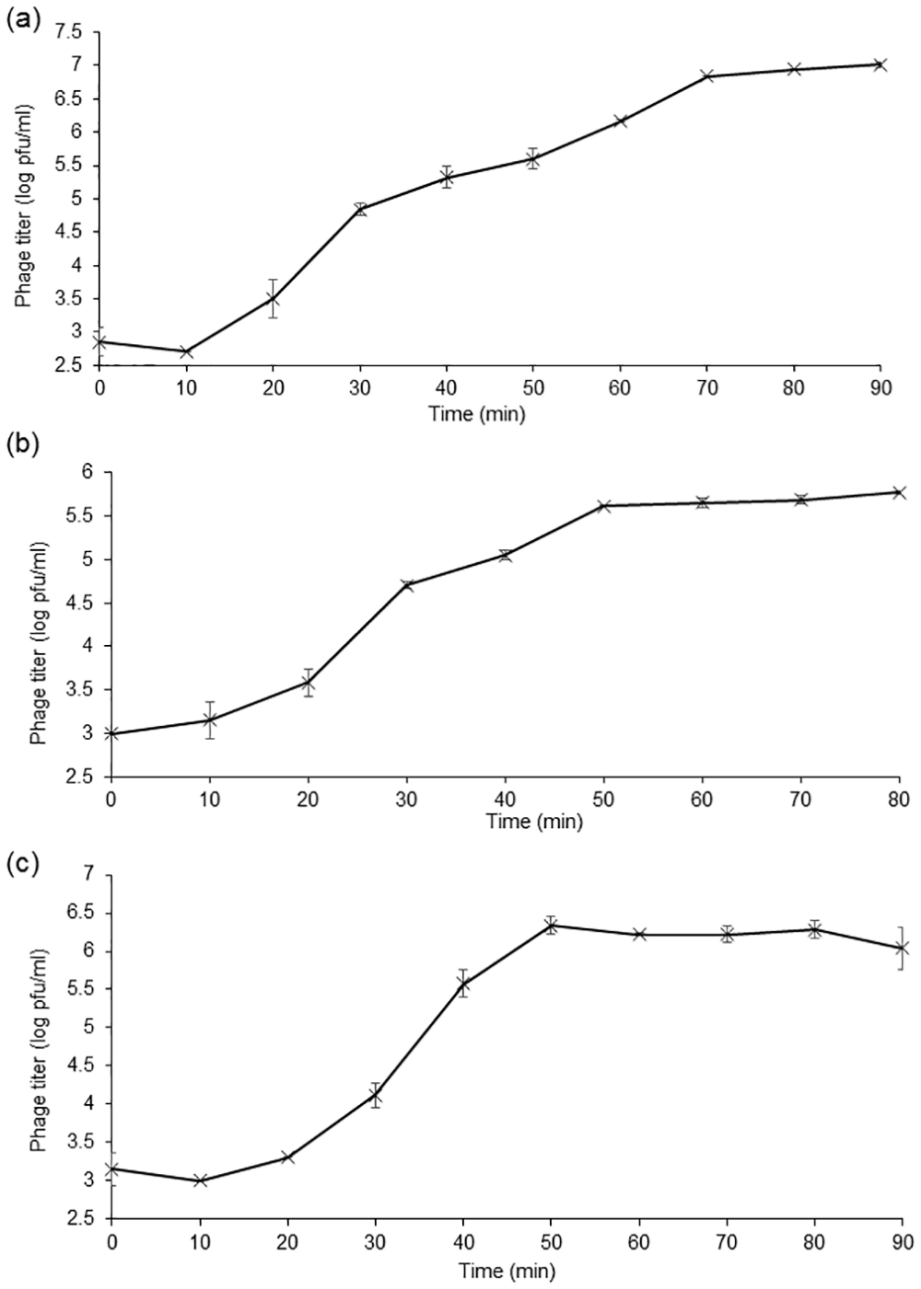
One-step growth curves of phages ɸAPCEc01 (a), ɸAPCEc02 (b), and ɸAPCEc03 (c) with *E*. *coli* strain DPC6051 in LB broth at 37°C.

One-step growth experiments were performed to assess the population kinetics of ɸAPCEc01, ɸAPCEc02 and ɸAPCEc03 in the presence of *E*. *coli* strain DPC6051 (Fig 2). All phages had a latent phase of 10 min. ɸAPCEc01 had a rise phase of 60 min (Fig 2a), and had a burst size of 90.3 ± 1.4 phage particles. These results are characteristic of *Myoviridae* phages, which display latent periods ranging from 20 to 120 min and burst sizes of up to 100 phage particles [48]. The lytic cycle of ɸAPCEc02 was characterized by a rise phase of 40 min (Fig 2b). Its calculated burst size was rather small with only 30.8 ± 1.9 phage particles. APCEc03 had population dynamics characteristic of phage EP23, another *E*. *coli Siphoviridae* phage [45], with a rise phase of 40 min, and a burst size of 47.4 ± 11.3 phage particles (Fig 2c).

### Key feature of the genomes

The length of the assembled genomes of ɸAPCEc01, ɸAPCEc02 and ɸAPCEc03 was 168,771 bp, 135,400 bp and 103,737 bp, respectively. The GC content of ɸAPCEc01, ɸAPCEc02 and ɸAPCEc03 was 37.7%, 43.6%, and 38.9%, respectively (Table 3). The GC content of these three phages is lower than the GC content of *E*. *coli* which is around 50% [17], suggesting that some elements of the phage genomes may have been acquired from other phages infecting hosts with a lower GC content or could have evolved from an ancestor that infected a host with a lower GC content.

**Table 3:** Genome features of phages ɸAPCEc01, ɸAPCEc02 and ɸAPCEc03.

	ɸAPCEc01	ɸAPCEc02	ɸAPCEc03
Length (bp)	168,771	135,400	103,737
No. of ORFs	272	219	151
No. of tRNAs	2	5	8
GC content (%)	37.7	43.6	38.9
A (%)	31.8	29	31.2
T (%)	30.5	27.5	29.9
C (%)	18.1	21.3	19
G (%)	19.5	22.3	19.9
% genome coding	94.7	89.1	85.9

ɸAPCEc01 genome had 94.7% (272 ORFs) coding regions whereas ɸAPCEc02 and ɸAPCEc03 had 89.1% (218 ORFs) and 85.9% (151 ORFs) coding regions, respectively (Table 3). For all phages, gene annotation did not identify known toxins or toxin-related pFAM domains. In addition, no genomic markers indicating a temperate lifestyle were found (S1, S2 and S3 Tables). These latter genetic characteristics make these phages suitable candidates for phage therapy purposes as the absence of lysogenic traits and virulence factors are mandatory for phages being considered as biocontrol agents [49].

### ɸAPCEc01 genome

**Fig 3:**
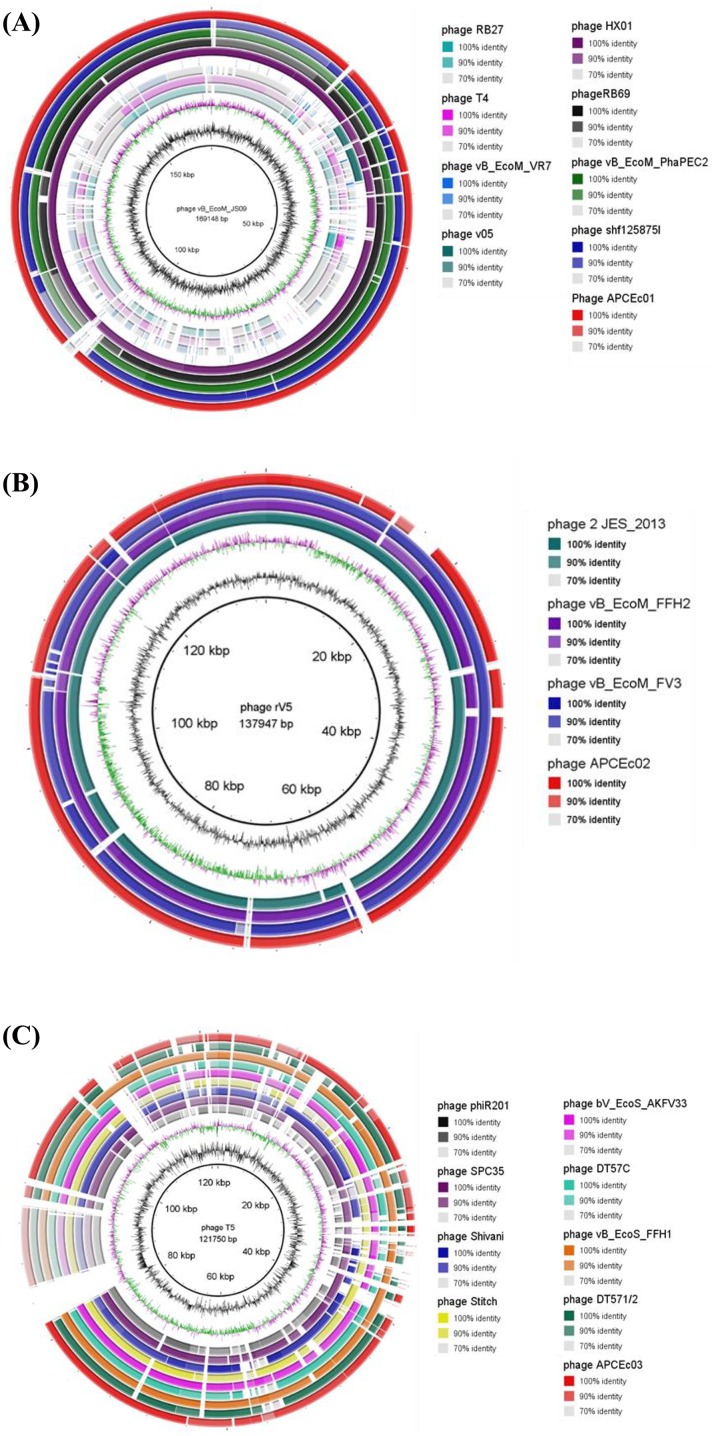
BLAST Ring Image Generator representation of phage ɸAPCEc01 (a), ɸAPCEc02 (b), and ɸAPCEc03 (c) genomes. The innermost rings show the GC content (black) and GC skew (purple: GC skew[-]; green: GC skew[+]). For each comparison using BRIG, the longest phage genome was used as a reference, and its name is indicated in the middle of the rings. The circles represent the genomes of the phages compared to this reference including the phages described in this study.

Phage ɸAPCEc01 genome is closely related at the nucleotide level to *Shigella* phage shf125875 (Fig 3a). This is in accordance with the fact that ɸAPCEc01 targets both *E*. *coli* and *Shigella* strains (Table 1). ɸAPCEc01 genome also shares at least 90% identity at the nucleotide level with four other T4-like *E*. *coli* phages, two of which (i.e., phages HX01 and vB_EcoM_PhAPEC2) have been used against avian pathogenic *E*. *coli* strains [50, 51] (Fig 3a). Of the 272 ORFs, 117 (43%) could be assigned a putative function. The conserved ORFs such as genes coding terminase, phage head and tail structural proteins, polymerase, and lysozyme show high identity hits with these phages (S1 Table). The T4-like phages have already been described as safe in numerous phage therapy applications [52], with no impact on commensal microbiota, while helping in the reduction of infection with *E*. *coli*.[53] A phage cocktail of at least ten T4 phages was even proposed to cover the five main *E*. *coli* pathotypes isolated from diarrhea patients [53]. This enhances the potential of phage ɸAPCEc01 for phage therapy purposes and the need to combine it with other phages in a phage cocktail.

### ɸAPCEc02 genome

The ɸAPCEc02 genome is closely related at the nucleotide level to four other rv5-like *E*. *coli* phages [38] (Fig 3b). The ORFs of ɸAPCEc02 share between 77 and 100% identity at the amino acid level with homologous ORFs of these four phages, with the exception of ORFs 65, 66, 208 and 209 (S2 Table). Of the identified 219 ORFs, only 50 (23%) could be assigned a putative function. This small number of ORFs with assigned functions emphasizes the need for the characterization of new phage genomes, which can only be achieved by the isolation and comparative analysis of new phages such as those in this study [54].

### ɸAPCEc03 genome

Of the 151 identified ORFs, 56 ORFS of ɸAPCEc03 (37%) could be assigned a putative function. With the exception of ORF38 coding for a homing endonuclease, all 56 ORFs of know functions share at least 87% identity at the amino acid level with homologues in twelve other phages, including four *E*. *coli* phages, three *Salmonella* phages and a *Yersinia* phage (S3 Table, Fig 3c). This is not unexpected, as the T5-like phage SPC35 was also found to target both *S*. *enterica* serovar Typhimurium and *E*. *coli* [55]. Despite these genetic similarities to other phage species genomes, ɸAPCEc03 did not lyse the *Salmonella* strains tested in this study, hence it would require to be tested against a broader range of strains to draw any conclusions as to its efficiency against species other than *E*. *coli*.

### Effect of phages on *E*. *coli* growing culture and biofilms

**Fig 4:**
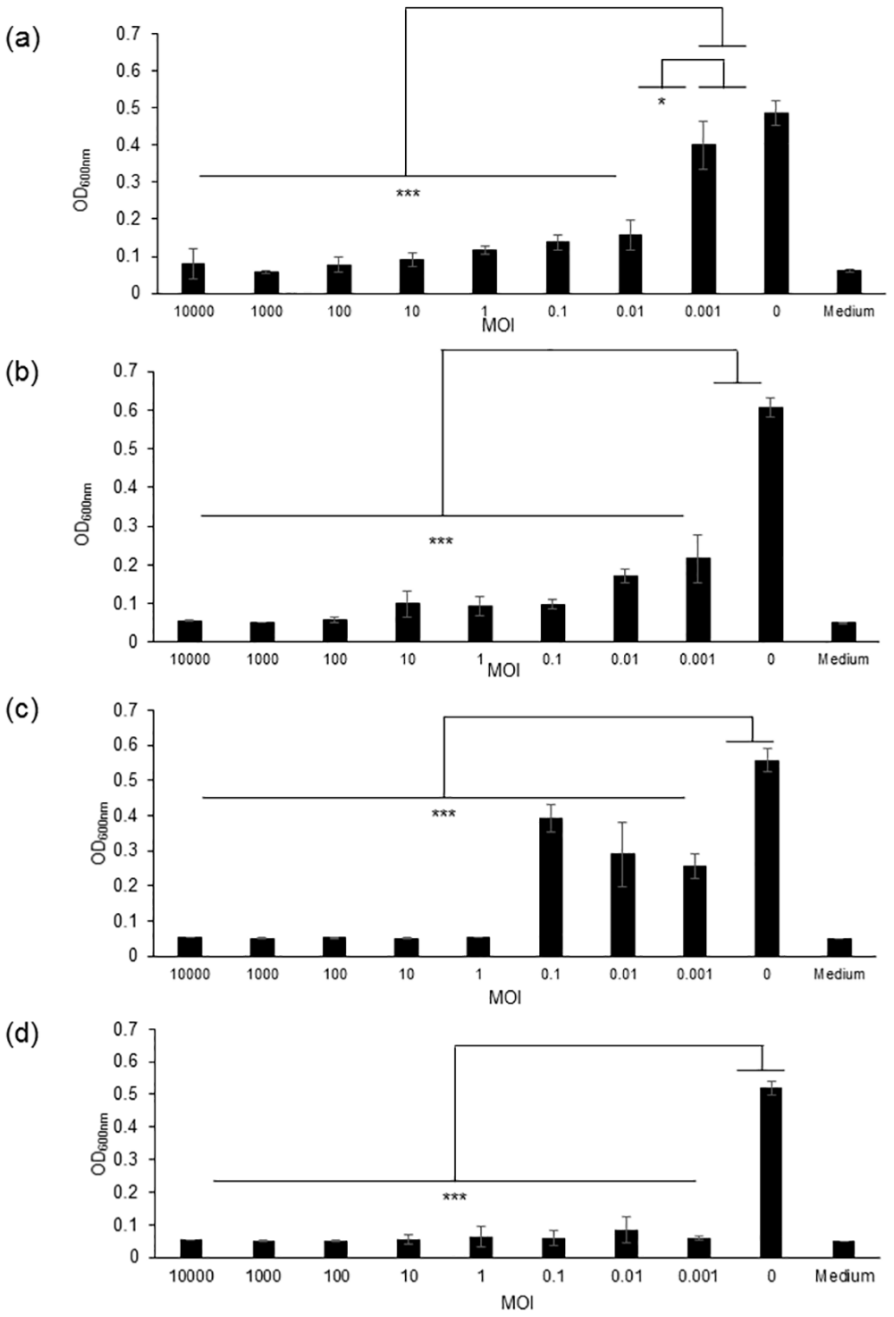
Bacterial challenge test. The optical density (OD_600nm_) was measured after 24 h of contact between *E*. *coli* strain DPC6051 and phage ɸAPCEc01 (a), phage ɸAPCEc02 (b), phage ɸAPCEc03 (c), and a cocktail of the three phages (d). *** p<0.001, * p<0.05.

As the genome features of the three phages did not exhibit any lysogenic or virulence factors, their potential as biocontrol agents against *E*. *coli* was evaluated. *E*. *coli* strain DPC6051 was sensitive to the three phages, and consequently was chosen as model strain in the subsequent part of the study. The effect of ɸAPCEc01, ɸAPCEc02 and ɸAPCEc03, alone or in cocktail, on the growth of strain DPC6051 was tested at different MOIs after 24 h of contact between the strain and the phages (Fig 4). The three phages significantly inhibited (p<0.001) or reduced (p<0.05) the growth of the *E*. *coli* strain at all tested MOIs (Fig 4a, 4b and 4c). This was especially true at MOI’s values greater than 1, for which the OD_600nm_ value of the culture was close to the OD_600nm_ value of the uninoculated broth. In the case of phage ɸAPCEc03, OD values seemed to increase with increasing MOIs (Fig 4c) for MOI’s values between 0.01 and 0.1. This increase in OD was however not significant (p>0.05) for the three values of MOI tested. A three-phage cocktail also inhibited the growth of the *E*. *coli* strain at all the tested MOIs (Fig 4d). These results prove the efficiency of the phages alone or in a cocktail to reduce the growth of *E*. *coli*.

**Fig 5:**
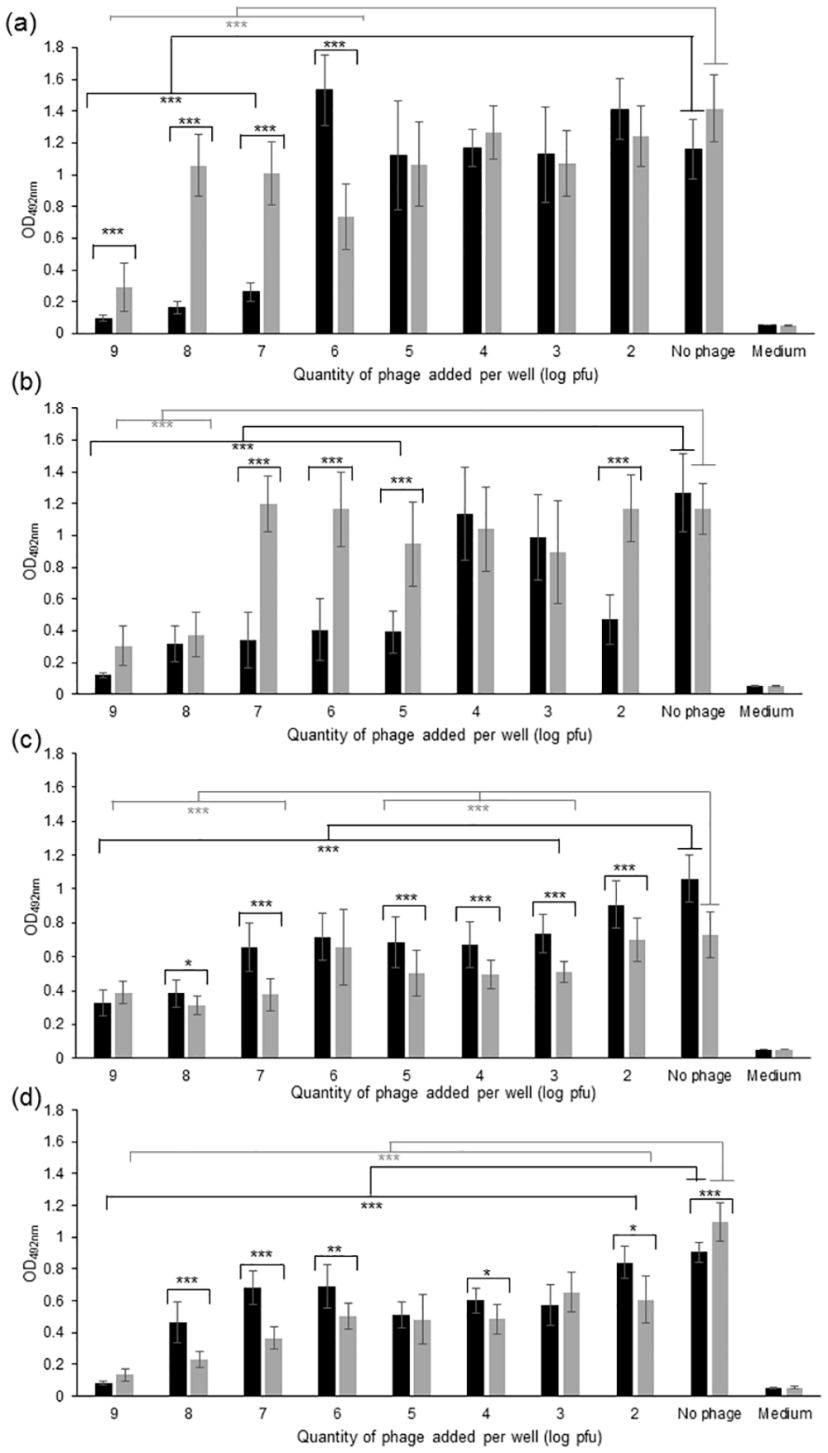
Effect of single phages ɸAPCEc01 (a), ɸAPCEc02 (b), ɸAPCEc03 (c), and a three-phage cocktail (d) on a 24 h-biofilm formed by *E*. *coli* strain DPC6051, after 24 h (■) and 48 h (■) of contact between phage and biofilm. Biofilm activity was assessed by OD_492nm_ measures after treatment with XTT supplemented with menadione. ***p<0.001; **p<0.01; *p<0.05.

*E*. *coli* is able to form and survive in biofilms, which control and prevention are a serious challenges in medicine [56]. In the current study, the action of phages alone and in cocktail against a biofilm formed by *E*.*coli* strain DPC6051 was assessed in a model system after 24 h and 48 h of contact with the phage (Fig 5). The metabolic activity of the cells forming the biofilm was measured using a tetrazolium salt (XTT) assay, XTT being only reduced by metabolically active cells to a coloured water-soluble formazan derivative quantified by colorimetry [26]. The activity of the biofilm was drastically reduced by at least 4-fold after 24 h of contact with phage ɸAPCEc01 at doses higher than 10^7^ plaque forming units (pfu)/well (Fig 5a). At lower doses, the biofilm activity was not reduced. After 48 h of contact with ɸAPCEc01, the metabolic activity of the biofilm significantly increased at all phage doses. This indicates the possible emergence of bacteriophage-insensitive mutants (BIMs) in the biofilm. The appearance of *E*. *coli* BIMs at a low frequency has been described in another bacterial challenge trial with single phages and phage cocktails [57]. The same tendencies at 24 h and 48 h were observed when the biofilm was in contact with ɸAPCEc02 (Fig 5b). The biofilm metabolic activity significantly decreased after 24 h of contact with ɸAPCEc03 (p< 0.001), and remained the same or even kept decreasing after another 48 h (Fig 5c). The phage cocktail at doses higher than 10^3^ pfu/well significantly reduced the biofilm metabolic activity by at least 1.2-fold (p<0.001) and almost eliminated all activity at 10^9^ pfu/well (Fig 5d). Another 48 h-incubation step in the presence of the phage cocktail again resulted in the maintenance or in a significant decrease in the biofilm metabolic activity (p<0.05). Based on these results, the phage cocktail represents a suitable biocontrol agent for the reduction and control of *E*. *coli* biofilms. It prevented the appearance of BIMs which are likely to occur with single phage [57], bearing in mind that phage activity also depends upon multiple factors such as the complex biofilm composition, the accessibility of the target bacteria within the biofilm and the environmental conditions [58].

### Combination of phage and antibiotic

**Fig 6:**
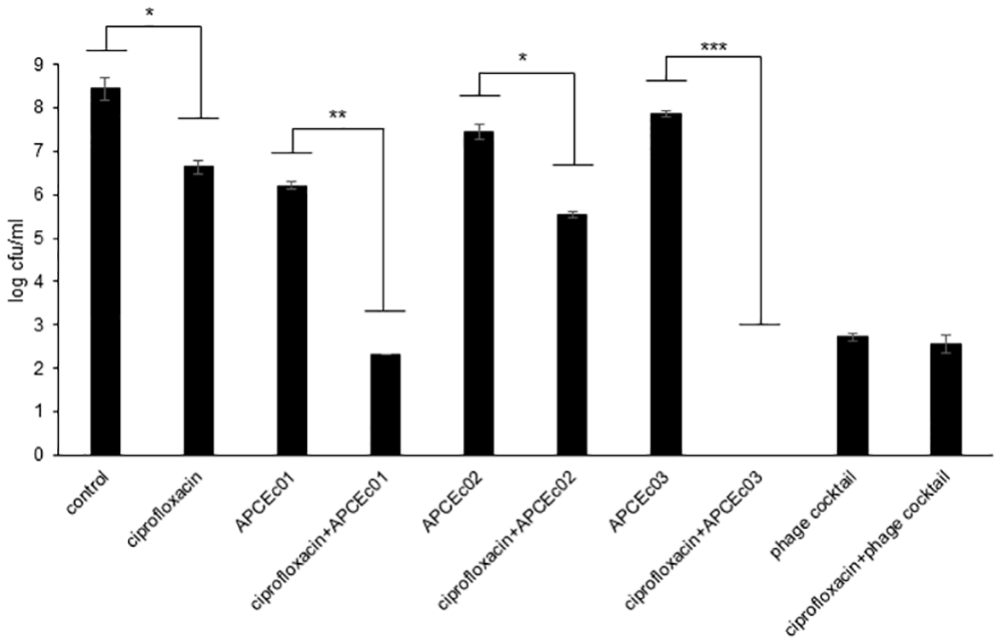
Effect of a combination of ciprofloxacin HCl and phages ɸAPCEc01, ɸAPCEc02, and ɸAPCEc03, alone or in cocktail, on the growth of *E*. *coli* strain DPC6051. Each condition was tested in triplicate. Bacterial counts were performed after 24 h of incubation, with a detection threshold of 20 cfu/ml. *** p<0.001, ** p<0.01, * p<0.05.

The combination of antibiotics and phage has been shown to efficiently reduce the growth of *E*. *coli*, and to greatly limit the emergence of antibiotic and phage resistant cells [59]. We investigated these aspects with the new coliphages when combined with ciprofloxacin. A growing culture of *E*. *coli* strain DPC6051 was incubated for 24 h at 37°C in the presence of a combination of ciprofloxacin (minimum inhibitory concentration (MIC) of 5 μg/ml) and phages ɸAPCEc01, ɸAPCEc02 and ɸAPCEc03, alone or in cocktail (MOI of 1) (Fig 6). The combination of antibiotic and phage decreased the number of live cells of 3.9 and 1.9 log colony forming units (cfu)/ml compared to phage ɸAPCEc01 and ɸAPCEc02 used alone, respectively, and of 4.3 and 1.1 log cfu/ml compared to antibiotic used alone. These results are in accordance with previous observations of phage-antibiotic synergy limiting the appearance of resistant mutants [60]. In the case of ɸAPCEc03, live cells could not be detected with the combination of antibiotic and phage, indicating the complete inhibition of emergence of antibiotic and phage resistant bacterial cells. These observations are congruent with previous results showing that antibiotics, such as ciprofloxacin, can be combined with phages to stimulate increased phage production and/or activity and thus improve the efficacy of bacterial killing [61]. Surprisingly, no significant synergetic effect of the antibiotic and the phage cocktail was observed. Nonetheless, the reduction in the number of live cells was of almost 6 log cfu/ml with the phage cocktail compared to the control. The phage cocktail also reduced the number of live cells of almost 4 log cfu/ml when compared to the use of ciprofloxacin alone. These three new phages are therefore suitable for usage in combination with antibiotics as they noticeably reduced the emergence of resistant cells when combined with ciprofloxacin. More antibiotics would need to be tested to evaluate the synergy range of the new phages with other antibiotics.

In conclusion, three new *E*. *coli* phages were isolated from human faeces samples in this study. These novel phages show promise as antimicrobial agents especially when used in a cocktail to prevent *E*. *coli* growth and biofilm formation. They can also be considered as complementary treatments to antibiotics by helping to prevent the appearance of resistant mutants. This study underlines the importance of mining the human gut for isolating and exploiting new antimicrobial agents such as phages [35].

## Supporting Information

S1 Table Ten first BLASTP hits of φAPCEc01 ORFs. (XLSX)

S2 Table Ten first BLASTP hits of φAPCEc02 ORFs. (XLSX)

S3 Table Ten first BLASTP hits of φAPCEc03 ORFs. (XLSX)
